# Identification of key proteins of cytopathic biotype bovine viral diarrhoea virus involved in activating NF-κB pathway in BVDV-induced inflammatory response

**DOI:** 10.1080/21505594.2022.2135724

**Published:** 2022-10-31

**Authors:** Wenlu Fan, Yixin Wang, Sheng Jiang, Yuan Li, Xin Yao, Mei Wang, Jinghua Zhao, Xiaobo Sun, Xiaoxia Jiang, Linhan Zhong, Yanyan Han, Houhui Song, Yigang Xu

**Affiliations:** a Key Laboratory of Applied Technology on Green-Eco-Healthy Animal Husbandry of Zhejiang Province, College of Animal Science & Technology, College of Veterinary Medicine, Zhejiang A&F University, Hangzhou, P.R. China; b Zhejiang Provincial Engineering Research Center for Animal Health Diagnostics & Advanced Technology, College of Animal Science & Technology, College of Veterinary Medicine, Zhejiang A&F University, Hangzhou, P.R. China; c College of Animal Science & Technology, Nanjing Agricultural University, Nanjing, P.R. China; d College of Veterinary Medicine, Northeast Agricultural University, Harbin, P.R. China

**Keywords:** Cytopathic biotype BVDV, inflammatory response, NF-κB pathway, Erns protein, NS5A protein

## Abstract

Bovine viral diarrhoea virus (BVDV) is the etiologic agent of bovine viral diarrhea-mucosal disease, one of the most important viral diseases in cattle, with inflammatory diarrhea, enteritis, and mucosa necrosis as the major clinical manifestations. NF-κB is an important transcription complex that regulates the expression of genes involved in inflammation and immune responses. NLRP3 inflammasome plays a key role in the development of inflammatory diseases. However, whether the activation of NF-κB is crucial for BVDV infection-induced inflammatory responses remains unclear. The results of our present study showed that BVDV infection significantly activated the NF-κB pathway and promoted the expression of NLRP3 inflammasome components (NLRP3, ASC, pro-caspase 1) as well inflammatory cytokine pro-IL-1β in BVDV-infected bovine cells, resulting in the cleavage of pro-caspase 1 and pro-IL-1β into active form caspase 1 and IL-1β. However, the levels of the NLRP3 inflammasome components and inflammatory cytokines were obviously inhibited, as well the cleavage of pro-caspase 1 and pro-IL-1β in the pre-treated bovine cells with NF-κB-specific inhibitors after BVDV infection. Further, cytopathic biotype BVDV (cpBVDV) Erns and NS5A proteins with their key functional domains contributed to BVDV-induced inflammatory responses via activating the NF-κB pathway were confirmed experimentally. Especially, the NS5A can promote cholesterol synthesis and accelerate its augmentation, further activating the NF-κB signalling pathway. Conclusively, our data elucidate that the activation of NF-κB signaling pathway plays a crucial role in cpBVDV infection-induced inflammatory responses.

## Introduction

Bovine viral diarrhoea virus (BVDV) is the etiologic pathogen of bovine viral diarrhea-mucosal disease (BVD-MD) with inflammatory diarrhea, enteritis, and mucosa necrosis as the major clinical manifestations. BVD-MD is one of the economically important diseases in cattle, leading to numerous losses to the cattle rearing industry all over the world [1–3]. BVDV, belonging to the genus *Pestivirus* within the family *Flaviviridae*, possesses a positive-sense single-stranded RNA genome (ca. 12.3 kb in length) encoding a large polyprotein, which can be proteolytically cleaved into structural proteins C, Erns, E1, and E2, and nonstructural proteins Npro, P7, NS2, NS3, NS4A, NS4b, NS5A, and NS5B [4,5]. BVDV affects cattle populations worldwide, causing immunologic suppression, enteritis, pneumonia diseases, and reproductive disturbance in cattle [6]. To date, underlying pathogenic mechanisms of BVDV are still not fully elucidated, and vaccines combined with eradication strategy contribute effectively to the control of BVDV infection [7,8].

Inflammation (diarrhoea, enteritis, and mucosa necrosis) is an extremely important event in the pathogenesis during BVDV infection. NF-κB, a key transcription mediator, plays a critical role in regulating the expression of genes involved in inflammatory and immune responses, apoptosis, and other stress responses [9–11]. The NF-κB is an evolutionarily conserved family, which is comprised of structurally related proteins working as homologous or heterologous dimers [12,13]. The members of the NF-κB family in mammalian cells include NF-κB1 (p50/p105), NF-κB2 (p52/p100), RelA (p65), c-Rel, and RelB. All these members possess a highly conserved domain called the Rel homology domain, which contains the DNA-binding domain, the NF-κB inhibitory protein (IκB)-interacting region, and the nuclear localization sequence [14]. Among these members, the RelB, c-Rel, and p65 contain transcriptional activation domains that positively regulate gene expression, but not in p50 and p52. The homodimer formed with itself is a transcriptional repression complex, which can inhibit gene transcription after binding to the target gene [15].

The activation of the NF-κB signalling pathway is marked by the nuclear translocation of active NF-κB. According to the species of IκB kinase (IKK) and NF-κB dimer involved in the NF-κB pathway, the NF-κB is activated mainly through two pathways, the canonical pathway and the non-canonical pathway. The canonical NF-κB pathway has been researched extensively, which can be triggered by many stimuli, such as virus, TNF-α, TLR ligands, LPS, and poly(I:C) [15,16]. Dissociation and degradation of the IκB from the complex is key to activating the canonical NF-κB signaling pathway. Firstly, the IκBα is phosphorylated by IKKβ, and then the phosphorylated IκBα is tagged by ubiquitination and subsequently degraded in proteasomes, leading to the release of the p65/p50 [15,17]. Then, the free p65/p50 is translocated into the nucleus to regulate the expression of target genes [18,19]. The non-canonical NF-κB pathway is a RelB/p52-mediated NF-κB pathway, which depends on the processing of the p100 to generate p52 [20,21]. Firstly, IKKα is activated by NF-κB-inducing kinase (NIK), and then the activated IKKα triggers phosphorylation of the p100 to generate the p52. Then, the NF-κB dimmers containing the p52 are translocated into the nucleus to mediate the expression of target genes [21–23].

Many viral infections can activate the NF-κB signalling pathway, and then regulate the transcription and expression of the target genes involved in host innate immunity and inflammatory responses [24,25]. In our previous studies, to explore the underlying mechanisms of BVDV-host interactions, we performed an integrative analysis of transcriptomics and proteomics of the BVDV-infected bovine cells, and found that the significantly differentially expressed genes and proteins were mainly enriched in the NF-κB, NOD-like receptors (NLRs), TNF, Toll-like receptors, and apoptosis signaling pathways [26,27]. We also found that the NLR protein 3 (NLRP3) inflammasome components (NLRP3, ASC, and pro-caspase 1) were significantly upregulated in BVDV-infected bovine cells. It is well known that the NLRP3 is the most studied inflammasome sensor involved in infection-triggered inflammation, and the NLRP3 inflammasome assembled from NLRP3, ASC, and pro-caspase 1 plays an important role in the development of inflammatory diseases, which can promote the self-cleavage of the pro-caspase 1 into its active form caspase 1, and then inflammatory cytokine pro-IL-1β is cleaved into its active form IL-1β by the activated caspase 1, and released from the cytoplasm, eventually causing host inflammatory responses [28,29]. BVDV infection can cause severe inflammation (diarrhoea, enteritis, and mucosa necrosis), and whether the activation of the NF-κB pathway is crucial for the NLRP3 inflammasome-mediated inflammatory response during BVDV infection remains unclear. In our present study, we investigated the activation of NF-κB signaling pathway induced by BVDV infection and its effect on the NLRP3 inflammasome-mediated inflammatory response, followed by the identification of the major viral proteins with their key functional domains involved in activating the NF-κB signaling pathway.

## Materials and methods

### Virus, cells, and reagents

Cytopathic biotype BVDV (cpBVDV) AV69 was kept in our laboratory. Bovine testicular (BT) cells and 293T cells kept in our laboratory were grown in DMEM (Gibco, USA) containing 10% foetal bovine serum (FBS) (Gibco, USA) at 37°C in a 5% CO_2_ incubator. The cpBVDV AV69 was propagated in BT cells. UV-inactivated BVDV was obtained by irradiating BVDV under ultraviolet light for 60 min, followed by detection using indirect immunofluorescence assay (IFA) with mouse anti-BVDV NS4B protein monoclonal antibody (mAb, prepared by our laboratory as a primary antibody) after blind passage for 3 generations. Mouse anti-HA, anti-β-actin mAbs were purchased from Abcam (USA). Rabbit polyclonal antibodies against NF-кB p65, phospho-NF-кB p65, IкBα, Lamin B were obtained from Affinity Biosciences (USA). Rabbit polyclonal antibodies against IL-1β, NLRP3, ASC, and caspase 1 were obtained from Proteintech (China).

### Plasmids

Plasmid pMD-19Ts purchased from TaKaRa (China) was used to clone the genes encoding cpBVDV strain AV69 structural proteins C, Erns, E1, and E2, and nonstructural proteins Npro, P7, NS2, NS3, NS4A, NS4b, NS5A, and NS5B. After that, these genes were subcloned into a eukaryotic expression plasmid pCMV-HA that was kept in our laboratory, generating the recombinant plasmids expressing these 12 proteins of cpBVDV AV69, respectively. Renilla luciferase reporter plasmid pRL-TK and NF-κB luciferase reporter plasmid pNF-κB-luc were obtained from Beyotime (China). The primers used for amplifying the genes that encode the proteins of cpBVDV AV69 were listed in Table 1.

**Table 1.** Primers used for amplifying the genes encoding BVDV proteins.

Gene	Primer sequences (5’→3’)	Product size
*C*	F: TCGGGCCATGGAGGCCTGCTCCGACACAAAT	309 bp
	R: CGCCTCGAGTTATCCCACTGCAACCTGAAACAAAACTAAGCC	
*E0*	F: TCGGGCCATGGAGGCCGAGAACATAACACAAT	681bp
	R: CGCCTCGAGTTATGCATATGCCCCAAACCATGTCTTACTCT	
*E1*	F: TCGGGCCATGGAGGCCGCCTCTCCCTACT	585 bp
	R: CGCCTCGAGTTACCCTTGTGCTCCTGTTATGAGT	
*E2*	F: TCGGGCCATGGAGGCCCTCCCAGCCTGTAAA	1122 bp
	R: CGCCTCGAGTTAACCTAAGGTCGTTTGTTCTGATATGATC	
*Npro*	F: TCGGGCCATGGAGGCCATGGAGTTGATCACAAAT	501 bp
	R: CGCCTCGAGTTAGCTTGAAACCCATAGAGGGCA	
*P7*	F: TCGGGCCATGGAGGCCGCCCAGTATGGGGCAGGTGAA	210 bp
	R: CGCCTCGAGTTATGCCCTTGCCATTCCTCC	
*NS2*	F: TCGGGCCATGGAGGCCGAACCAGGCGCCCAGGGCTA	1404 bp
	R: CGCCTCGAGTTATCTTAAGATCCATCCTAGGTGTT	
*NS3*	F: TCGGGCCATGGAGGCCGGGCCTGCCGTGTGCAAAAAAAT	2049 bp
	R: CGCCTCGAGTTACAGTCCTACCACTTGCTTCAGTGCTTTCCC	
*NS4A*	F: TCGGGCCATGGAGGCCTCTACTGCTGAGAAT	192 bp
	R: CGCCTCGAGTTATAACTCCTTTAGTTCAGTCTCTTTCCCCTC	
*NS4B*	F: TCGGGCCATGGAGGCCGCAGTGGGTGACTTGGACAAGATTAT	1041 bp
	R: CGCCTCGAGTTACAGGTTCCTTATCTTCCCTTCAGAGTCCATCC	
*NS5A*	F: TCGGGCCATGGAGGCCTCTGGGAATTATATCTT	1488 bp
	R: CGCCTCGAGTTACAGCTTCATTGTGTAGGTCC	
*NS5B*	F: TCGGGCCATGGAGGCCTCTAGTTGGTTTCTTC	2185 bp
	R: CGCCTCGAGTTACCTGCTGGCACCGACGGCT	

The restriction enzyme sites are marked by underlined letters.

### Transfection

To detect the activation of the NF-κB pathway induced by BVDV infection, BT cells (over 80% confluent in 24-well plates) were transfected with the pNF-κB-luc reporter plasmid (0.44 μg/well) and internal reference plasmid pRL-TK (0.06 μg/well) using lipofectamine® LTX & Plus Reagent (Invitrogen, USA). At 12 hours after transfection, the transfected BT cells were infected with cpBVDV AV69 at a multiple of infection (MOI) of 1.0, using the UV-inactivated BVDV as control. The cell samples were collected at 12 h, 24 h, and 48 h after BVDV infection, followed by the determination of the firefly luciferase and the Renilla luciferase activities using a Dual-Luciferase® Reporter Assay System (Promega, USA). To screen the key viral proteins and the key functional domains involved in activating the NF-κB signalling pathway, BT cells or 293T cells (over 80% confluent in 24-well plates) were co-transfected with recombinant eukaryotic plasmids expressing BVDV proteins (or truncated proteins), pNF-κB-luc, and pRL-TK, using lipofectamine® LTX & Plus Reagent (Invitrogen, USA), followed by the determination of the NF-κB relative dual-luciferase activities. In parallel, co-transfection with empty plasmid pCMV-HA was used as negative control. All reporter gene assays were performed in triplicates and repeated thrice. Data are presented as the mean ± SD values.

### Indirect immunofluorescence (IFA)

Cell samples with different treatments (BVDV infection, transfection or cholesterol) were collected, washed twice with PBS (pH = 7.2), and then were fixed with 4% of paraformaldehyde at room temperature (RT) for 30 min, followed by washing twice with PBS. Subsequently, the cells were treated with 0.2% of Triton X-100 for permeabilization at RT for 10 min, followed by blocking with 0.3% of bovine serum albumin (BSA) at 37°C for 1 h. Then, the cells were incubated separately with the primary antibodies at 37°C for 1 h, and then incubated with diluted secondary antibodies (FITC-labelled goat anti-mouse/rabbit IgG antibody or TRITC-labeled goat anti-mouse IgG antibody) (Abcam, USA) at 37°C for 1 h. After treating with DAPI (Sigma, USA) for 15 min and washing with PBS, the protein expression or subcellular localization was observed by fluorescence microscope or laser confocal microscope.

### Quantitative real-time reverse transcription PCR (qRT-PCR)

At 12 h, 24 h, and 48 h after BVDV infection, total RNA of BVDV-infected BT cells and mock-infected BT cells was respectively extracted using TRIzol reagent (Invitrogen, USA), and then was subjected to reverse transcription to generate cDNA with *M*-MLV Reverse Transcriptase (TaKaRa, China). Using the cDNA as the template, the changes in expression levels of the target genes in the BVDV-infected bovine cells were determined by a SYBR Green-based qRT-PCR. In parallel, the pre-treated BT cells with NF-κB-specific inhibitor (Bay 11–7082 and QNZ) that were infected with BVDV were used as a control, and BT cells treated with DMSO were used as a negative control. In addition, the total RNA of the bovine cells transfected with recombinant eukaryotic plasmids expressing the NF-κB-activating key viral proteins or truncated proteins was extracted and reverse transcribed into cDNA, and then the changes in expression levels of the genes (*NLRP3*, *pro-caspase-1*, *ASC*, and *pro-IL-1β*) were determined, using *β-actin* as an internal reference. The primers used for qRT-PCR were listed in Table 2.

**Table 2.** Primers used for qRT-qPCR.

Gene	Primer sequences (5’→3’)
*β-actin*	F: GCCAACCGTGAGAAGATGAC
	R: AGGCATACAGGGACAGCACA
*IL-6*	F: CACTGACCTGCTGGAGAAGATGC
	R: CCGAATAGCTCTCAGGCTGAACTG
*IL-8*	F: TGCCTGTTGAACTGCGCCTTG
	R: AGTGCTTCCACATGTCCTCACATC
*IL-1β*	F: ATGAAGAGCTGCATCCAACACCTG
	R: ACCGACACCACCTGCCTGAAG
*TNF-α*	F: CTGGCGGAGGAGGTGCTCTC
	R: GGAGGAAGGAGAAGAGGCTGAGG
*COX-2*	F: TGGTCTGGTGCCTGGTCTGATG
	R: TGTCTGGAACAACTGCTCATCGC
*NOS3*	F: ACCATCCTGTACGCCTCTGAGAC
	R: ACCAGTGCCTCGTGCTCCAG
*NLRP3*	F: AAGAAGCTCTGGTTGGTCAGTTGC
	R: GGAATGGTTGGTGCTCAGGACAG
*Caspase-1*	F: GCTTGCATCTTCAGGACCAGGAG
	R: CAACATCAGCTCCGTCTCTTCTGG
*ASC*	F: AGAGGAGCAGTACCAGGCAGTG
	R: CCAGGTCGTCCACCAGGTAGG

### Western blotting

To prepare the protein samples for Western blotting analysis: the total cellular proteins were extracted by Total Protein Extraction Kit (Promega, USA); the nuclear proteins were extracted by Nuclear Protein Extraction Kit (Beyotime, China); the cytoplasmic proteins were extracted using Cytoplasmic Protein Extraction Kit (Beyotime, China). All protein samples were quantified by a BCA Protein Assay Kit (Beyotime, China) prior to SDS-PAGE, followed by mixing with 5×SDS loading buffer and boiling for 10 min. Next, the proteins separation was performed via SDS-PAGE. Then, the electrophoresed proteins were transferred onto a PVDF membrane (Millipore, USA), followed by blocking overnight at 4°C with 5% skim milk in PBS containing 0.1% of Tween-20 (PBST). After washing thrice with PBST buffer, the PVDF membrane was incubated overnight at 4°C with the primary antibodies, and then incubated with diluted horseradish peroxidase-labelled goat anti-mouse/rabbit IgG (Abcam, USA) at 37°C for 1 h, followed by the visualization of immunoblots using a SuperEnhanced Chemiluminescence Detection Kit (Applygen Technologies Inc., China).

### Determination of cholesterol level

Cell samples were collected from normal cells (mock group), BVDV-infected cells group, pCMV-NS5A-transfected cells group, pCMV-PKS_ER-transfected cells group, and pCMV-transfected cells group (negative control group), followed by the determination of cholesterol level using the Amplex^TM^ Red Cholesterol Assay Kit (Invitrogen, USA) according to the manufacturer’s instructions. Briefly, the cells were washed thrice with PBS, and incubated in PBS (pH 7.4) containing 2% TritonX-100 (Sigma, USA) at 37°C for 30 min, followed by the treatment with Good’s Buffer containing cholesterol esterase, phenol, 4-AAP, cholesterol oxidase, and peroxidase at 37°C for 10 min. Next, the absorbance of each cell sample was measured at 510 nm by SpectraMax ABS Absorbance Microplate Reader (Molecular Devices, USA), and the cholesterol content (mmol/L) was calculated as the following formula: (sample OD value-blank OD value/standard sample OD value-blank OD value) × standard sample content.

### Statistical analysis

In this study, data are shown as the mean (columns) ± standard deviation, SD (bars). Data analysis for the differences among groups was performed by Tukey’s multiple-comparison tests and one-way analysis of variance using GraphPad Prism V8.0 (GraphPad, USA).

## Results

### BVDV infection-induced NF-κB signalling pathway activation

**Figure 1.**
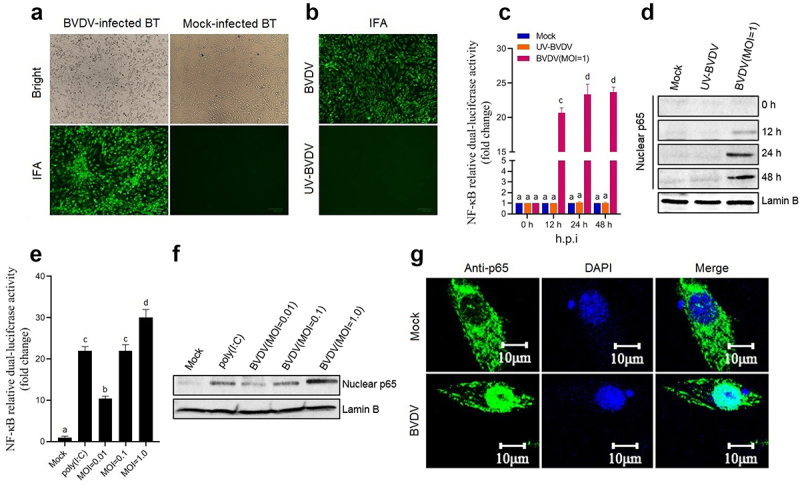
Activation of the NF-κB signalling pathway induced by BVDV. A: Detection of BVDV-infected BT cells by IFA; B: Detection of UV-inactivated BVDV-infected BT cells by IFA, using live BVDV as control; C: The NF-κB relative dual-luciferase activity in BVDV-infected BT cells determined at 12 h, 24 h, and 48 h after BVDV infection, using mock- and UV-inactivated BVDV-infected cells as control; D: Detection of the p65 nuclear translocation at 12 h, 24 h, and 48 h after BVDV infection by Western blotting; E: Determination of relationship between NF-κB relative dual-luciferase activity and viral dose; F: Detection of relationship between the p65 nuclear translocation and viral dose; G: Detection of BVDV-induced p65 nuclear translocation by IFA. Data are given as means ± SD values. The lowercase letters (a vs. b; b vs. c; c vs. d) indicate significant difference of *P* <0.05; a vs. c and b vs. d indicates significant difference of *P* <0.01; a vs. d indicates significant difference of *P* <0.001.

In this study, BT cell was used as a cell model to evaluate the NF-κB pathway activation triggered by BVDV infection. As shown in Figure 1A, the cpBVDV AV69 was effectively propagated in BT cells. Using UV-inactivated BVDV as control (Figure 1B), the NF-κB relative dual-luciferase activities in the transfected BT cells was determined at different time points after BVDV infection. Results showed that significant luciferase activity was detected in the BVDV-infected BT cells (*P* < 0.001 or *P* < 0.0001) (Figure 1C), compared with UV-inactivated BVDV group and mock group, indicating that the NF-κB was activated after BVDV infection. The activation of the NF-κB was further confirmed by Western blotting analysis that the nuclear translocation of the p65 was induced by BVDV infection (Figure 1D), displaying a dose-dependent manner (Figure 1E and 1F). Moreover, IFA results showed that the nuclear translocation of the p65 was observed in the BVDV-infected BT cells, but not observed in UV-inactivated BVDV group (Figure 1G). Our data demonstrated that live cpBVDV infection can effectively induce the nuclear translocation of the p65 to activate the NF-κB pathway.

### BVDV infection-induced inflammatory response depends on the NF-κB pathway activation

**Figure 2.**
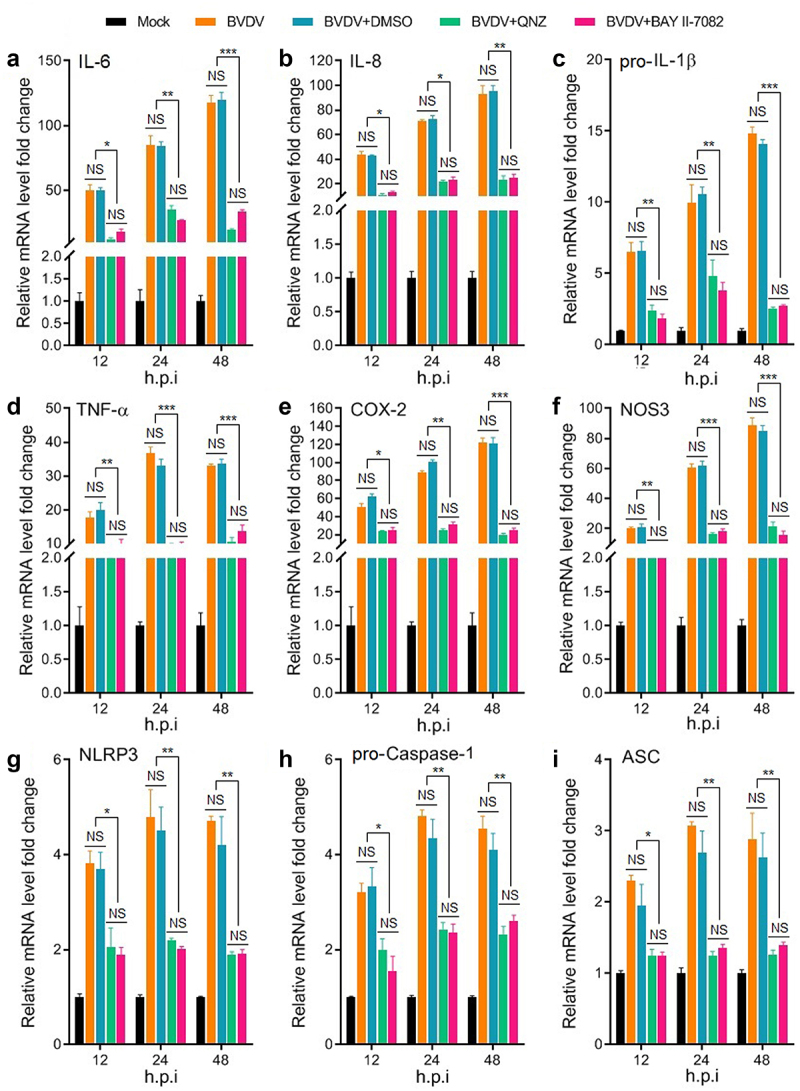
Expression levels of the genes encoding cytokines (IL-6, IL-8, pro-IL-1β, TNF-α, COX-2, and NOS-3) and NLRP3 inflammasome (NLRP3, pro-caspase 1, and ASC) determined by qRT-PCR. Total RNA of BT cells from mock group, BVDV group, BVDV+DMSO group, BVDV+BAY 11–7082 (NF-κB inhibitor) group, and BVDV+QNZ (NF-κB inhibitor) group was extracted and reverse transcribed into cDNA respectively, followed by qRT-PCR detection. Results indicated that BVDV infection-induced inflammatory response was regulated by the activation of NF-κB signalling pathway. Data are given as means ± SD values. *, *P* <0.05; **, *P* <0.01; ***, *P* <0.001.

**Figure 3.**
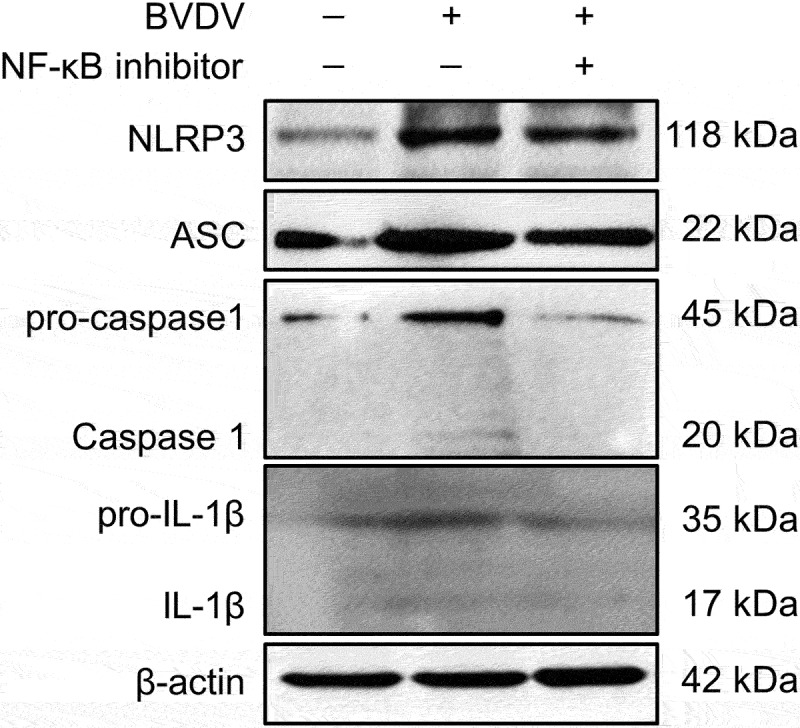
Identification of BVDV infection-induced NLRP3 inflammasome activation and the cleavage of pro-caspase 1 and pro-IL-1β by Western blotting, using β-actin as an internal reference.

In order to analyse whether BVDV infection-induced inflammatory response is mediated by the activation of the NF-κB signalling pathway, the mRNA levels of the genes encoding inflammatory cytokines IL-6, IL-8, COX-2, NOS-3, TNF-α, and pro-IL-1β, and NLRP3 inflammasome components (NLRP3, pro-caspase-1, and ASC) were determined by qRT-PCR in mock-infected cells group, BVDV-infected cells group, BVDV+DMSO cells group, BVDV+QNZ (NF-κB inhibitor) cells group, and BVDV+BAY 11–7082 (NF-κB inhibitor) cells group, respectively. The results showed that BVDV infection significantly promoted the expression of these genes, while the expression of the genes were significantly inhibited in the bovine cells pre-treated with the NF-κB inhibitor after BVDV infection (Figure 2). Moreover, we determined the protein expression levels of the NLRP3 inflammasome components (NLRP3, ASC, and pro-caspase 1) and pro-IL-1β in the BVDV-infected bovine cells by Western blotting assay, and found that BVDV infection effectively induced the expression of NLRP3, ASC, pro-caspase 1, and pro-IL-1β, as well the cleavage of pro-caspase 1 and pro-IL-1β into their active form caspase 1 and IL-1β. However, the protein levels of the NLRP3, ASC, pro-caspase 1, and pro-IL-1β were significantly inhibited in the NF-κB-specific inhibitor-treated bovine cells, as well the cleavage of pro-caspase 1 and pro-IL-1β (Figure 3). Our data demonstrate that BVDV infection can activate the NLRP3 inflammasome and induce IL-1β maturation, in which the production of NLRP3 inflammasome components and pro-IL-1β is regulated by the activation of the NF-κB pathway.

### Erns and NS5A mainly contributed to BVDV-induced inflammatory response via activating the NF-κB signalling pathway

**Figure 4.**
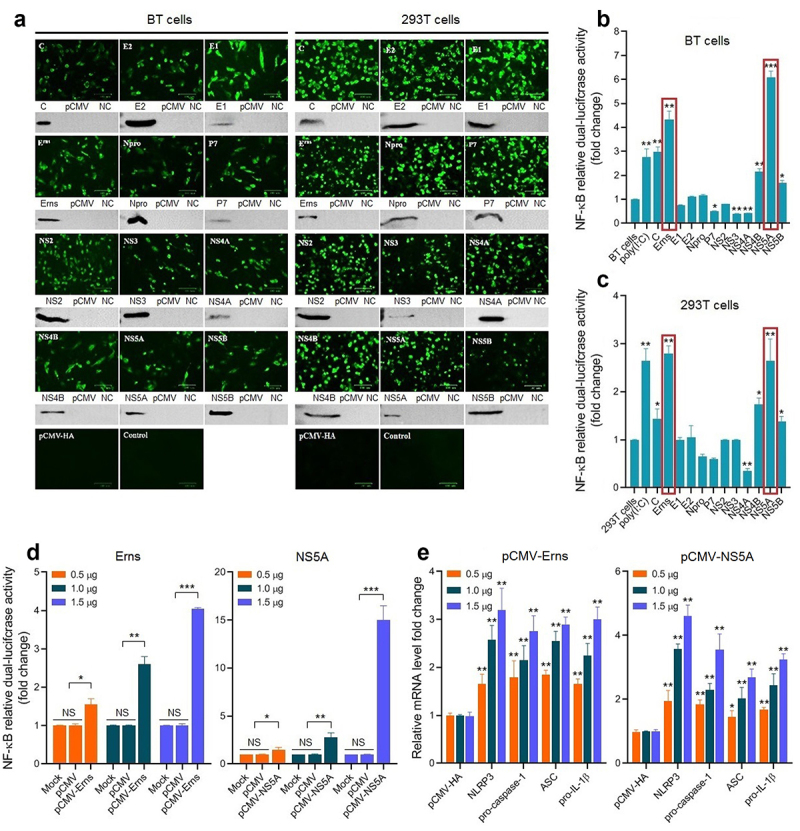
Expression and identification of BVDV proteins in BT cells and 293T cells transfected with recombinant eukaryotic plasmids expressing viral proteins by IFA and Western blotting (A). Viral proteins-induced NF-κB relative dual-luciferase activities in BT cells (B) and 293T cells (C) were detected after transfection, showing that the Erns and NS5A proteins mainly contributed to the activation of the NF-κB signalling pathway in a dose-dependent manner in BT cells (D). The expression levels of the genes encoding NLRP3, pro-caspase-1, ASC, and pro-IL-1β were determined in transfected bovine cells with the Erns-expressing plasmid and the NS5A-expressing plasmid (E), showing a dose-dependent manner. Data are given as means ± SD values. *, *P* <0.05; **, *P* <0.01; ***, *P* <0.001.

To screen the key viral proteins involved in activating the NF-κB signalling pathway during BVDV infection, the recombinant eukaryotic plasmids expressing BVDV proteins were transiently transfected into BT cells and 293T cells, followed by IFA and Western blotting identification (Figure 4A). Then, the NF-κB relative dual-luciferase activities were detected in the transfected BT cells (Figure 4(b)) and 293T cells (Figure 4C). The results showed that the Erns and NS5A proteins of cpBVDV mainly contributed to the activation of the NF-κB pathway, displaying a dose-dependent manner in BT cells (Figure 4D). We found that the expression levels of *NLRP3*, *pro-caspase-1*, *ASC*, and *pro-IL-1β* genes in the transfected bovine cells with the pCMV-Erns and pCMV-NS5A significantly increased in a dose-dependent manner (*P* < 0.01), compared with empty plasmid pCMV-HA group (Figure 4E). Our data suggest that cpBVDV Erns and NS5A proteins are the key viral proteins contributed to the activation of the NF-κB signal pathway in BVDV infection-induced inflammatory response.

### Erns and NS5A promoted IκBα degradation and p65 nuclear translocation

**Figure 5.**
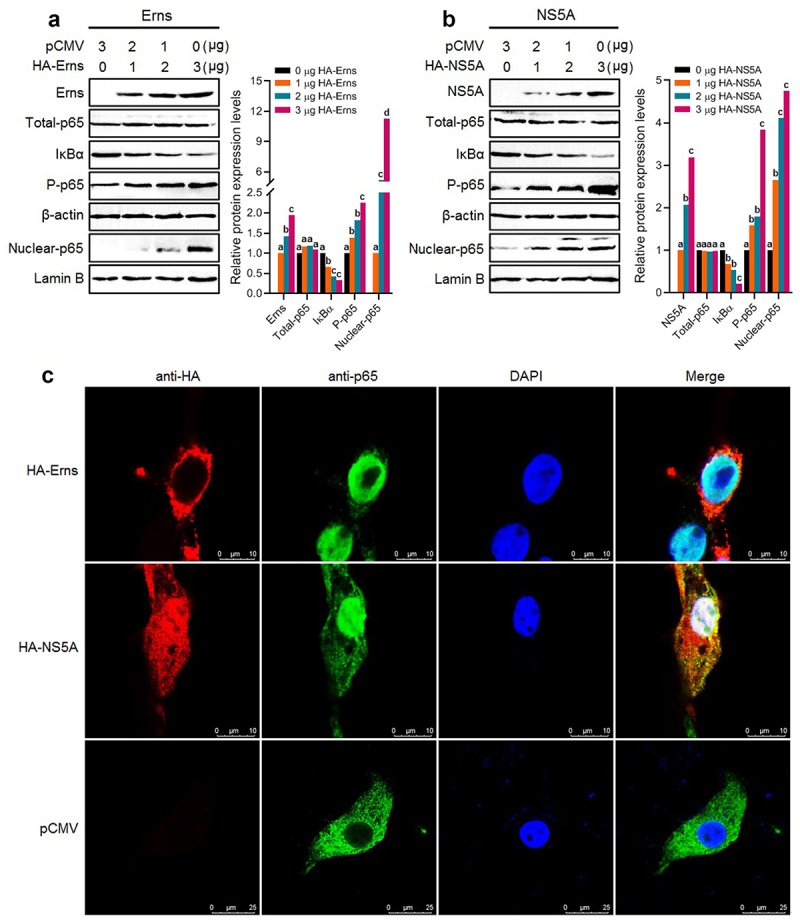
The IκBα degradation and the p65 phosphorylation in the cytoplasm and p65 nuclear translocation were detected in the transfected bovine cells with the Erns-expressing plasmid (A) and the NS5A-expressing plasmid (B) by Western blotting. C: The p65 nuclear translocation induced by the Erns and NS5A proteins was detected by IFA. The lowercase letters (a vs. b; b vs. c; c vs. d) indicate significant difference of *P* <0.05; a vs. c indicates significant difference of *P* <0.01; a vs. d indicates significant difference of *P* <0.001.

Degradation of the IκBα and nuclear translocation of the p65 are the key hallmarks of activating the NF-κB signalling pathway. In this study, BT cells were transfected separately with the plasmid pCMV-Erns expressing BVDV Erns protein and the plasmid pCMV-NS5A expressing BVDV NS5A protein, followed by determining the levels of total p65 in the cell lysates, IκBα and phosphorylated p65 (*P*-p65) in the cytoplasm by Western blotting using β-actin as an internal reference, and the level of p65 in the nucleus (nuclear p65) using Lamin B as an internal reference. The results showed that there were no significant changes in the protein levels of total p65 in the cells before and after transfection with the pCMV-Erns (Figure 5A) and pCMV-NS5A (Figure 5B), while the levels of the P-p65 in the cytoplasm and the nuclear p65 significantly increased, and the level of the IκBα in the cytoplasm decreased markedly after transfection with pCMV-Erns (Figure 5A) and pCMV-NS5A (Figure 5B), exhibiting a dose-dependent manner. Moreover, the IFA results showed that the nuclear translocation of the p65 was observed in the bovine cells transfected with the pCMV-Erns and the pCMV-NS5A, but not observed in the pCMV-HA-transfected bovine cells (Figure 5C). Out data demonstrate that the Erns and NS5A proteins of BVDV induce the nuclear translocation of the p65.

### Identification of key functional domains of the Erns and NS5A proteins involved in activating the NF-κB pathway

**Figure 6.**
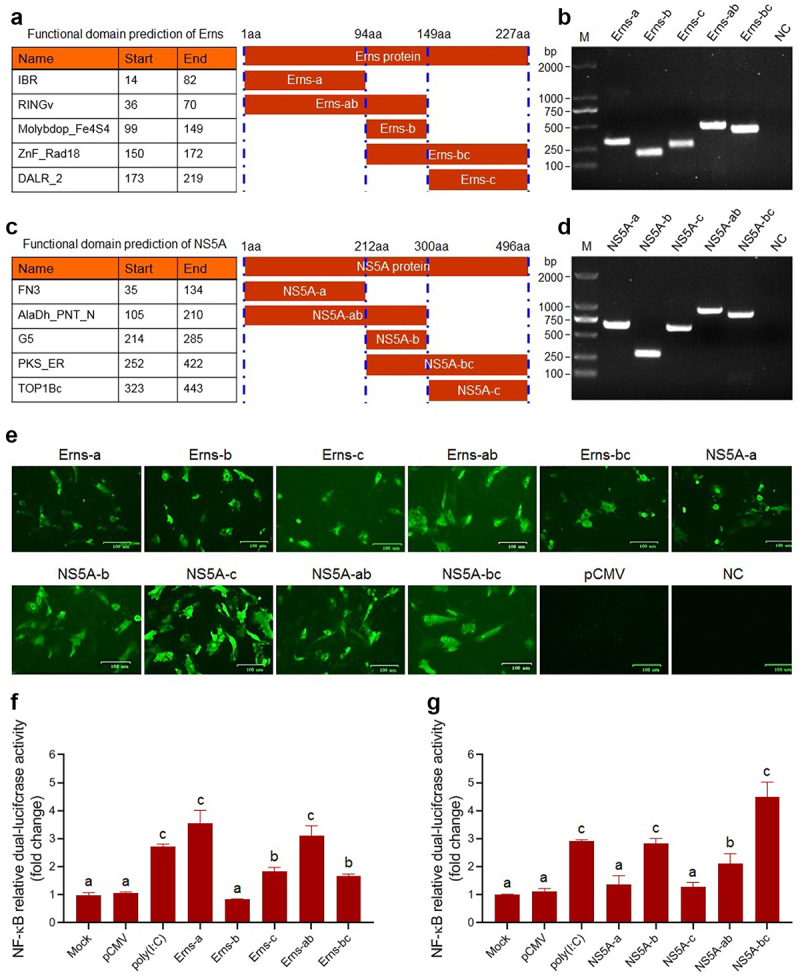
The prediction result of functional domains of the Erns protein (A) and NS5A protein (C) and amplification of the gene fragments encoding truncated Erns protein (B) and truncated NS5A protein (D). E: Expression and identification of truncated proteins in the transfected BT cells by IFA. F and G: The NF-κB relative dual-luciferase activities in the transfected BT cells with recombinant eukaryotic plasmids expressing truncated proteins. Data are given as means ± SD values. The lowercase letters (a vs. b; b vs. c) indicate significant difference of *P* <0.05; a vs. c indicates significant difference of *P* <0.01.

**Figure 7.**
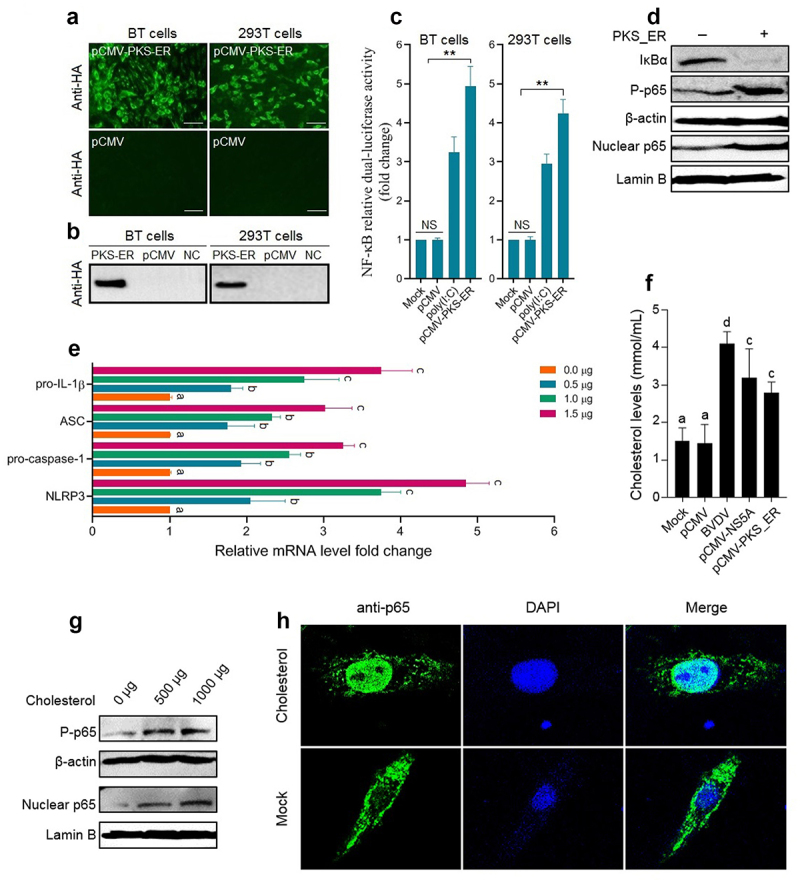
Identification results of the NF-κB pathway activation induced by the PKS_ER functional domain of the NS5A protein. Expression of PKS_ER functional domain in the transfected BT cells and 293T cells identified by IFA (A) and Western blotting (B). C: PKS_ER functional domain-induced NF-κB relative dual-luciferase activities detected in the transfected BT cells and 293T cells. D: PKS_ER functional domain-induced activation of the NF-κB pathway in the transfected bovine cells identified by Western blotting. E: The expression levels of the genes encoding NLRP3, pro-caspase-1, ASC, and pro-IL-1β were determined in the transfected bovine cells, showing a dose-dependent manner. F: Detection of the cholesterol levels in BVDV-infected bovine cells, NS5A-expressing bovine cells, and PKS_ER-expressing bovine cells, using normal bovine cells and pCMV-transfected bovine cells as the negative control. G: Identification of cholesterol-induced p65 phosphorylation and p65 nuclear translocation by Western blotting. H: Identification of cholesterol-induced p65 nuclear translocation by IFA. Data are given as means ± SD values. *, *P* <0.05; **, *P* <0.01; ***, *P* <0.001. The lowercase letters (a vs. b; b vs. c; c vs. d) indicate significant difference of *P* <0.05; a vs. c indicates significant difference of *P* <0.01; a vs. d indicates significant difference of *P* <0.001.

To identify the key functional domains of the Erns and NS5A proteins involved in activating the NF-κB signalling pathway, we predicted the functional domains of the Erns protein (Figure 6A) and the NS5A protein (Figure 6C) of BVDV via the SMART Website. Then, the gene fragments encoding truncated Erns protein (Figure 6B) and truncated NS5A protein (Figure 6D) were amplified by RT-PCR, followed by the construction of recombinant eukaryotic plasmids expressing truncated proteins and identification of the truncated proteins expression in the transfected BT cells by IFA (Figure 6E). Then, the truncated proteins-induced NF-κB relative dual-luciferase activities were detected, using poly(I:C) as a positive control. The results showed that the truncated proteins Erns-a, and Erns-c significantly activated the NF-κB, compared with mock group and pCMV group (*P* < 0.05 or *P* < 0.01) (Figure 6F), indicating that the IBR and the DALR_2 functional domains of the Erns protein contribute to the activation of the NF-κB, particularly the IBR functional domain (Figure 6A). As shown in Figure 6G, the truncated proteins NS5A-b, NS5A-ab, and NS5A-bc significantly activated the NF-κB, compared with mock group and pCMV group (*P* < 0.05 or *P* < 0.01). Based on the above results, we speculated that the PKS_ER functional domain (Figure 6C) was the key functional domain of NS5A protein contributed to the activation of NF-κB pathway. Thus, we constructed a recombinant plasmid pCMV-PKS_ER expressing PKS_ER functional domain to further confirm experimentally. The expression of PKS_ER functional domain in the transfected BT cells and 293T cells was identified by IFA (Figure 7A) and Western blotting (Figure 7B). Then, the PKS_ER functional domain-induced NF-κB activation was detected in the transfected BT cells and 293T cells, followed by the determination of the levels of *NLRP3*, *ASC*, *pro-caspase-1*, and *pro-IL-1β* genes. The results showed that the NF-κB relative dual-luciferase activities in the transfected BT cells and 293T cells increased significantly (*P* < 0.01), compared to mock cells group and pCMV-transfected cells group (Figure 7C). In addition, Western blotting results further confirmed that the PKS_ER functional domain of the NS5A protein can induce the IκBα degradation, the p65 phosphorylation and its nuclear translocation (Figure 7D). Our data demonstrate that the NF-κB pathway can be activated by the PKS_ER functional domain. The qRT-PCR results showed that the levels of the *NLRP3*, *pro-caspase-1*, *ASC*, and *pro-IL-1β* in the pCMV-PKS_ER-transfected bovine cells significantly increased in a dose-dependent manner (Figure 7E). Subsequently, we respectively determined the cholesterol levels in BVDV-infected bovine cells, pCMV-NS5A-transfected bovine cells, and pCMV-PKS_ER-transfected bovine cells, using mock bovine cells and pCMV-transfected bovine cells as the negative control. The results showed that cholesterol levels in BVDV-infected bovine cells, pCMV-NS5A-transfected bovine cells, and pCMV-PKS_ER-transfected bovine cells significantly increased (*P* < 0.01 or *P* < 0.001), compared to the control groups (Figure 7F), indicating that BVDV infection promotes cholesterol production in host cells. We also found that cholesterol can induce the p65 phosphorylation detected by Western blotting (Figure 7G) and the p65 nuclear translocation detected by Western blotting (Figure 7G) and IFA (Figure 7H), indicating that cholesterol can activate the NF-κB pathway.

## Discussion

Bovine viral diarrhoea-mucosal disease caused by BVDV is one of the most complex viral diseases in cattle, with diarrhea, acute and chronic enteritis, mucosa necrosis, and pneumonia as the main clinical symptoms. It is well known that the NF-κB pathway plays a key role in inflammatory responses, and the NLRP3 inflammasome plays an important role in the development of inflammatory diseases. Therefore, the aim of this study was to investigate whether BVDV-induced inflammatory responses was mediated through the activation of NF-κB pathway involved in inflammatory cytokines production.

Previous studies have reported that the NF-κB pathway-mediated inflammatory responses play a crucial role in the pathogenesis of many viral infections, such as porcine transmissible gastroenteritis virus [24], hepatitis C virus (HCV) [30], rabies virus [31], human coronavirus [32], and porcine epidemic diarrhoea virus [33]. BVDV exists two biotypes, namely cytopathic biotype BVDV (cpBVDV) and non-cytopathic biotype BVDV (ncpBVDV) [34]. Among the two biotypes of BVDV, it has been confirmed experimentally that the cpBVDV can activate the NF-κB pathway, but not ncpBVDV [35,36]. In our present study, we utilized the BT cell as a cell model to evaluate the ability of cpBVDV AV69 to activate the NF-κB pathway using NF-κB bioactivity-guide dual-luciferase reporter system. Our data demonstrated that the cpBVDV AV69 can effectively activate the NF-κB pathway in a dose-dependent manner, and promote the nucleus translocation of p65, which was consistent with previous studies [35,36].

Generally, inflammation is an important protective response for the body against virus invasion. It is well known that pathogen-associated molecular patterns and damage-associated molecular patterns can trigger inflammatory responses via host pattern recognition receptors [37,38]. On the one hand, the members of interferon regulator factor family are activated to regulate type I interferon antiviral immune response. On the other hand, the NF-κB pathway is activated to promote production of various inflammatory cytokines, such as IL-1β, IL-6, IL-8, and TNF-α, causing inflammatory responses. To date, researchers have improved our understanding of many inflammasome complexes such as the NLRP3, NLRC4, RIG-I, and AIM2. Among them, the NLRP3 inflammasome composed of NLRP3, pro-caspase-1, and ASC has been researched extensively, which can be effectively activated by viral infection, leading to the oversecretion of the cytokines involved in inflammatory responses (such as IL-1β, IL-18) and promoting the development of inflammatory diseases [39–42]. Studies have reported that classical swine fever virus (CSFV) and HCV, belonging to the family *Flaviviridae* same as BVDV, can effectively activate NLRP3 inflammasome [43,44]. In this study, our data showed that BVDV infection significantly promoted the expression of the genes encoding NLRP3, pro-caspase-1, ASC, and pro-IL-1β in the BVDV-infected bovine cells, and induced the cleavage of pro-caspase 1 and pro-IL-1β into their active form caspase 1 and IL-1β, indicating that BVDV infection can induce the NLRP3 inflammasome-mediated inflammatory response. Our work is under way to investigate BVDV-induced pyroptosis, including the NLRP3 inflammasome assembly and activation, GSDMD cleavage, and secretion of mature IL-1β. In addition, we also found that the mRNA levels of the genes encoding other inflammatory cytokines (IL-6, IL-8, COX-2, NOS-3, and TNF-α) increased remarkably after BVDV infection. However, the expression levels of all above genes in the BT cells pre-treated with the NF-κB-specific inhibitor was downregulated dramatically after BVDV infection, as well the protein levels of NLRP3, pro-caspase-1 and its cleavage, ASC, pro-IL-1β and its cleavage. Our data indicated that BVDV infection-induced inflammatory response was mediated through the activation of NF-κB signalling pathway. Interestingly, several other studies have shown that UV-inactivated virus still can activate inflammatory response, such as human cytomegalovirus [45]

Proteins are the basic functional units and the ultimate executors of biological functions. Studies have reported that many viruses may utilize their proteins to regulate the activation of the NF-κB pathway, such as TGEV [24], HCV [30,46], Human coronavirus [32], and PRRSV [47]. In this study, using the Dual-Luciferase Reporter Assay System, the viral proteins NS5A, Erns, C, NS4B, and NS5B were found to be potential contributors for activation of the NF-κB pathway, particularly the NS5A and Erns proteins. Studies have reported that the NS5A protein of BVDV and HCV played a similar role during viral infection [48,49]. It has been confirmed experimentally that HCV NS5A protein is able to activate the NF-κB pathway in HCV-infected cells [50–52]. Thus, to further confirm our findings in this study, the transfection of bovine cells with the NS5A-expressing or Erns-expressing eukaryotic plasmid showed that the IκBα degradation, the p65 phosphorylation and the p65 nuclear translocation were significantly induced by the Erns and NS5A protein in a dose-dependent manner, indicating that BVDV NS5A and Erns proteins can activate the NF-κB via canonical pathway. On this basis, the IBR functional domain located at 14aa − 82aa of Erns protein and the PKS_ER functional domain located at 252aa − 422aa of NS5A protein involved in activating the NF-κB pathway were further confirmed experimentally. Previous studies have reported that HCV NS5A protein can promote cholesterol synthesis [53], and cholesterol can trigger the NF-κB pathway activation involved in inflammation [54–58]. In addition, a study by researchers reported that BVDV NS5A protein mainly localized on the endoplasmic reticulum in the BVDV-infected cells [59]. Thus, we speculated that BVDV NS5A protein can promote the cholesterol production that may contribute to the activation of the NF-κB pathway, which was confirmed by us in this study. Interestingly, several studies have elucidated that cholesterol can activate the NLRP3 inflammasome [60,61]. Therefore, we will continue to explore the underlying mechanisms of BVDV infection-induced inflammatory responses via the activation of the NF-κB pathway.

**Figure 8.**
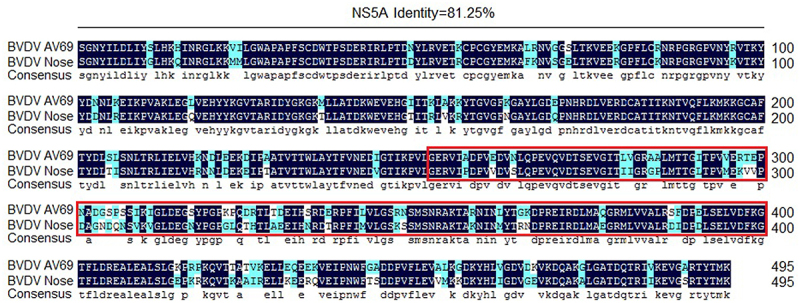
Analysis of the amino acid homology of the NS5A protein from BVDV strain AV69 and BVDV strain Nose.

However, Zahoor et al [59]. reported that BVDV NS5A protein could inhibit the activation of the NF-κB pathway in HEK293 and LB9.K cells, which was determined by luciferase reporter-gene assay. So, to analyse the underlying causes attributed to the difference in regulating the NF-κB pathway activation between the NS5A protein of BVDV strain AV69 used in this study and the NS5A protein of BVDV strain Nose (AB078951) reported by Zahoor et al [59], we analysed the amino acid homology of the NS5A protein from BVDV strain AV69 and BVDV strain Nose. We found that the amino acid homology of the NS5A protein was 81.25% between BVDV strain AV69 and BVDV strain Nose (Figure 8), while the amino acid homology within the PKS_ER functional domain at 252aa − 422aa of the NS5A was only 74.27%. Therefore, we speculate that the changes of key amino acids of NS5A lead to the alteration in protein function of regulating the NF-κB pathway activation, while this requires further experimental validation.

**Figure 9.**
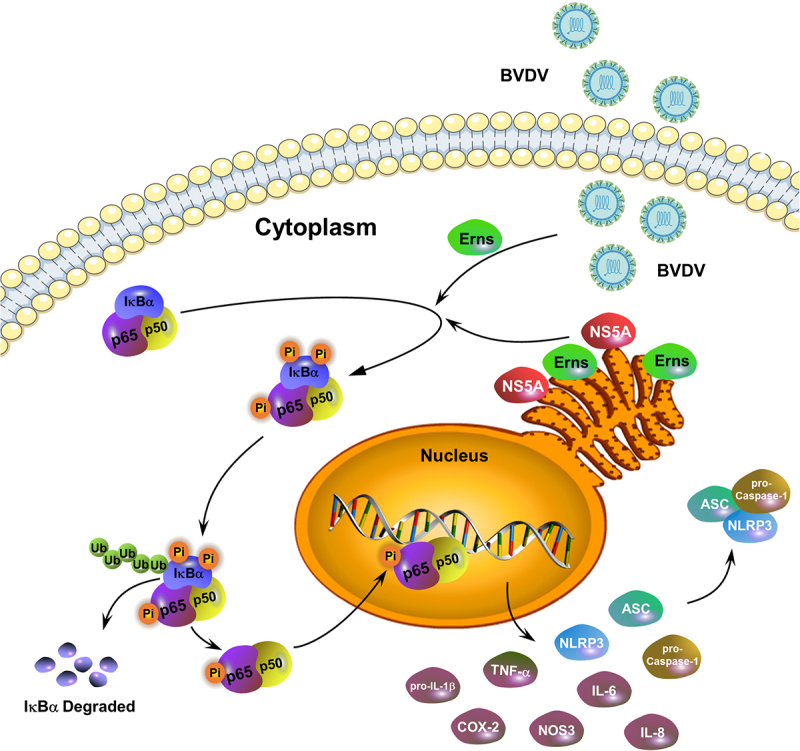
A schematic diagram of BVDV infection-induced inflammatory responses via the activation of the NF-κB pathway.

A schematic diagram of BVDV infection-induced the activation of the NF-κB signalling pathway was given in Figure 9. Conclusively, our results demonstrated that cpBVDV-induced inflammatory response was regulated by the activation of the NF-κB signaling pathway, followed by the identification of the key viral proteins and their key functional domains involved in activating the NF-κB pathway, suggesting that the viral proteins Erns and NS5A play a key role in the NF-κB-mediated inflammatory response induced by cpBVDV infection.

## Data Availability

The authors confirm that the data supporting the findings of this study are available within the article.
